# Supplementary material for: Mouse WIF1 Is Only Modified with O-Fucose in Its EGF-like Domain III Despite Two Evolutionarily Conserved Consensus Sites
Source: Biomolecules. 2020 Aug 28;10(9):1250. doi: 10.3390/biom10091250 (PMC7565927; doi:10.3390/biom10091250)
Supplement: Supplementary file 1 [file biomolecules-10-01250-s001.pdf]

SUPPLEMENTARY DATA

Mouse WIF1 Is Only Modified with *O*-Fucose in Its EGF-like Domain III Despite Two Evolutionarily Conserved Consensus Sites

Florian Pennarubia [1,2], Emilie Pinault [1,3], Bilal Al Jaam [1], Caroline E. Brun [1,4], Abderrahman Maftah [1,†,*], Agnès Germot [1,†] and Sébastien Legardinier [1,†]

[1] Glycosylation and cell differentiation, PEIRENE, EA 7500, Faculty of Sciences and Technology, University of Limoges, F-87060 Limoges, France; florian.pennarubia@uga.edu (F.P.); emilie.pinault@unilim.fr (E.P.); bilal.al.jaam@hotmail.com (B.A.J.); caroline.brun@univ-lyon1.fr (C.E.B.); agnes.germot@unilim.fr (A.G.); sebastien.legardinier@unilim.fr (S.L.)

[2] Complex Carbohydrate Research Center, University of Georgia, Athens, GA 30602, USA

[3] Mass Spectrometry Platform, BISCEm, US 042 INSERM - UMS 2015 CNRS, Faculty of Medecine and Pharmacy, University of Limoges, F-87025 Limoges, France

[4] NeuroMyoGene Institute, CNRS UMR 5310, INSERM U1217, University of Claude Bernard Lyon 1, 69008 Lyon, France

* Correspondence: abderrahman.maftah@unilim.fr; Tel.: +33555457684

† A.M., A.G. and S.L. are considered co-last authors and contributed equally to this work.

SUPPLEMENTARY DATA

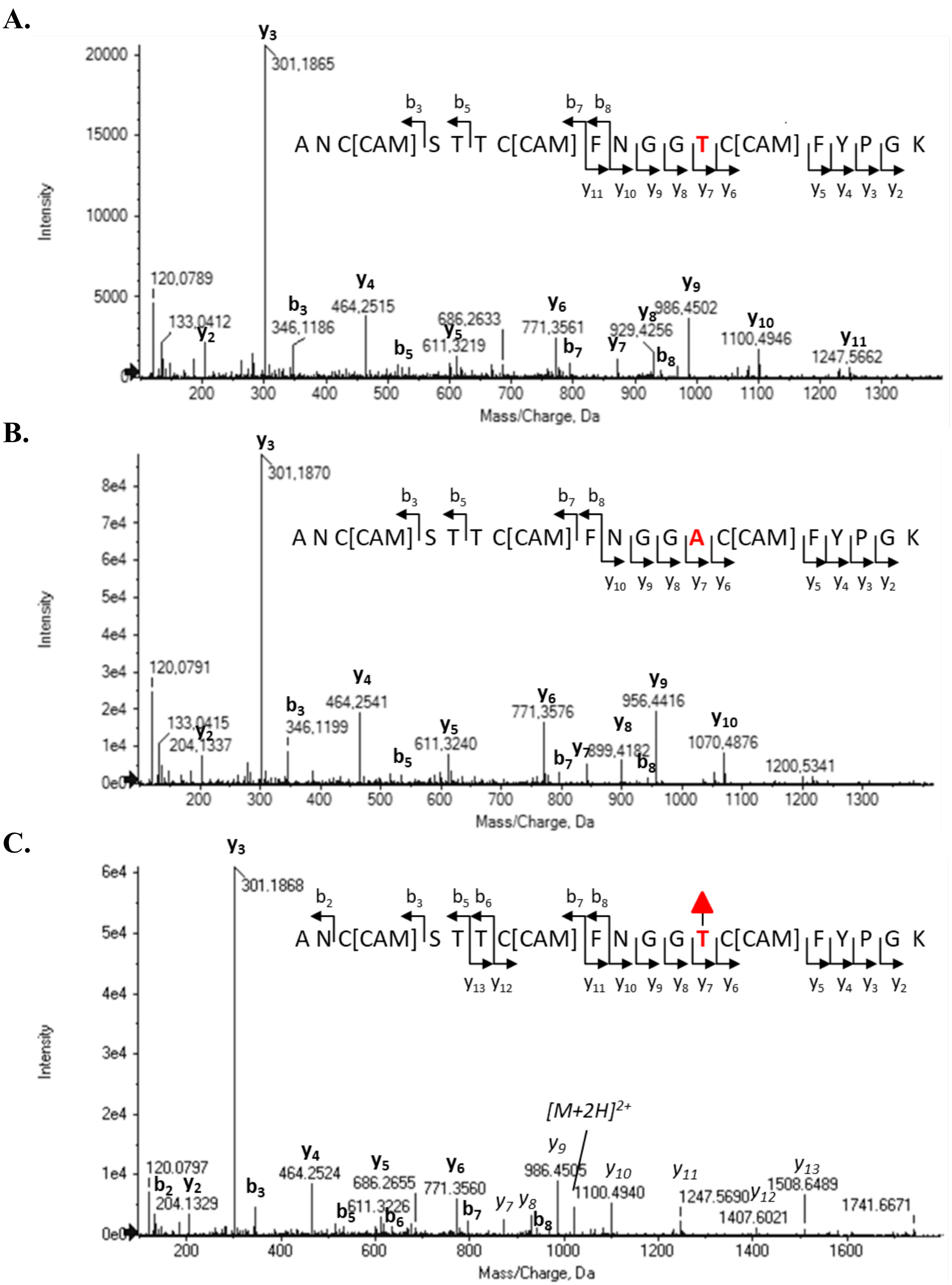

Figure S1. Representative MS/MS spectra of the peptide of interest from WT and T255A EGF-LD III digested with trypsin, after incubation with recPOFUT1 and GDP-fucose. Spectra, acquired with a micro-LC TripleTOF system in IDA mode, are shown for non-modified WT (**A**) and T255A (**B**) isolated EGF-LD III but also for WT EGF-LD III modified with *O*-fucose on its T^{255} (**C**). Detected b/y fragments are annotated on each spectrum confirming the identified peptide sequence. The presence of non-modified y fragments, indicated in italic (C), reflects the loss of *O*-fucose moeity which had been transferred. The threonine T^{255} of the *O*-fucosylation consensus site is indicated in red bold, even when mutated in alanine.

SUPPLEMENTARY DATA

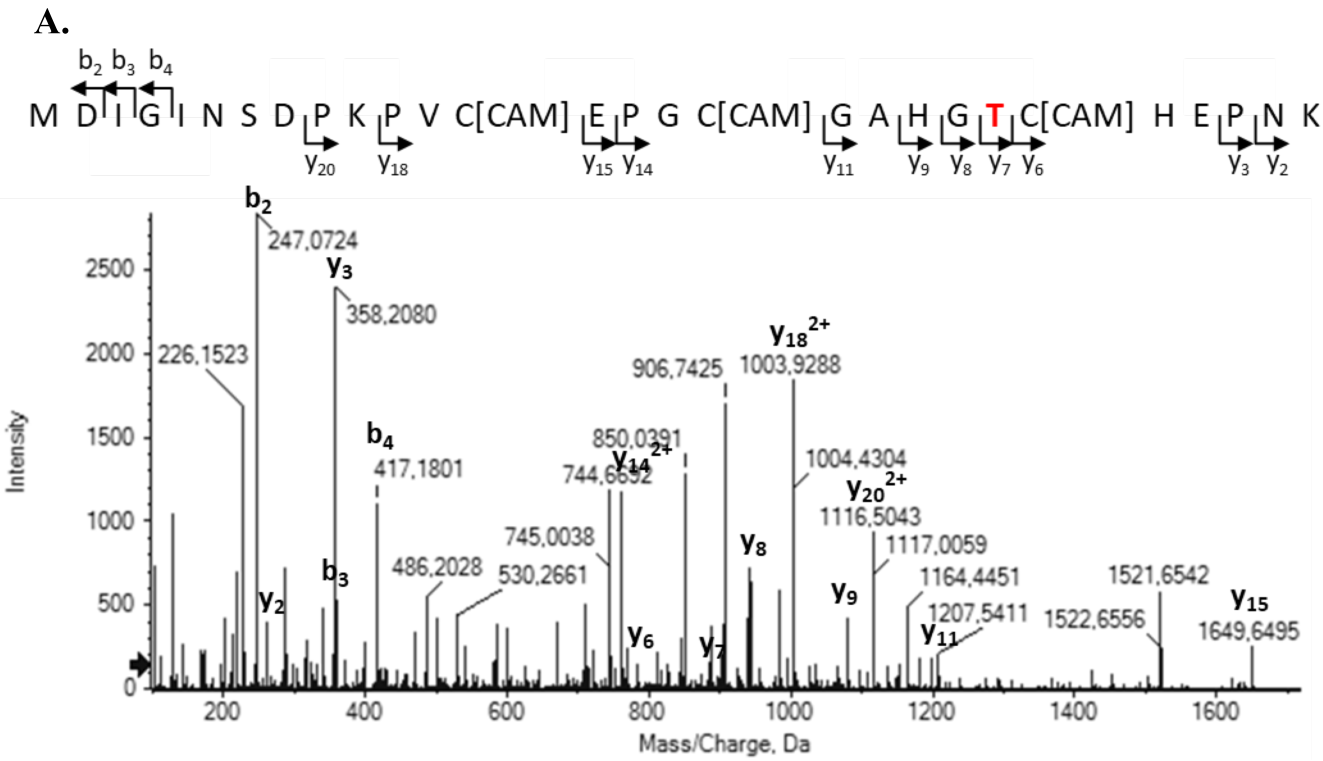

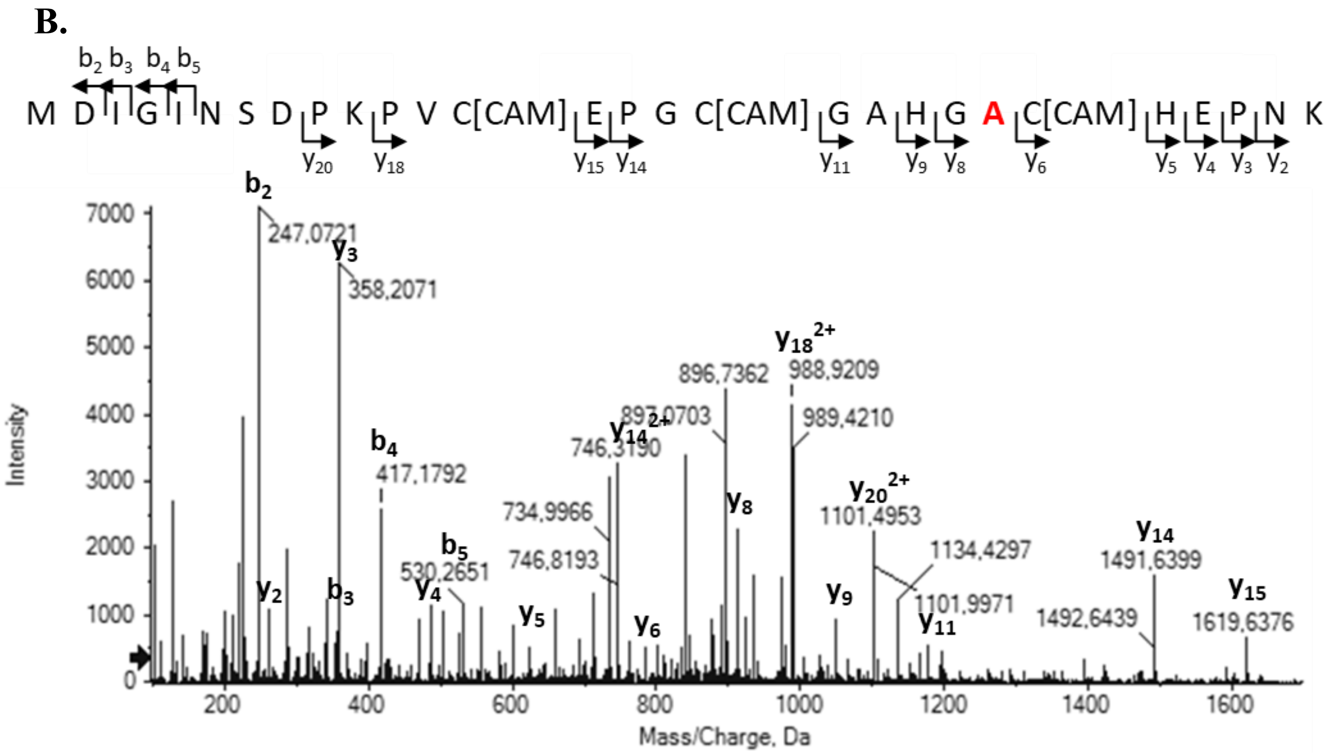

Figure S2. Representative MS/MS spectra of the peptide of interest from WT (A) and T319A (B) EGF-LD V digested with trypsin, after incubation with recPOFUT1 and GDP-fucose. Spectra were acquired with a micro-LC TripleTOF system in IDA mode. Detected b/y fragments are annotated on each spectrum, thus confirming the identified peptide sequence. The threonine T^{319} of the *O*-fucosylation consensus site is indicated in red bold, even when mutated in alanine.

SUPPLEMENTARY DATA

A.

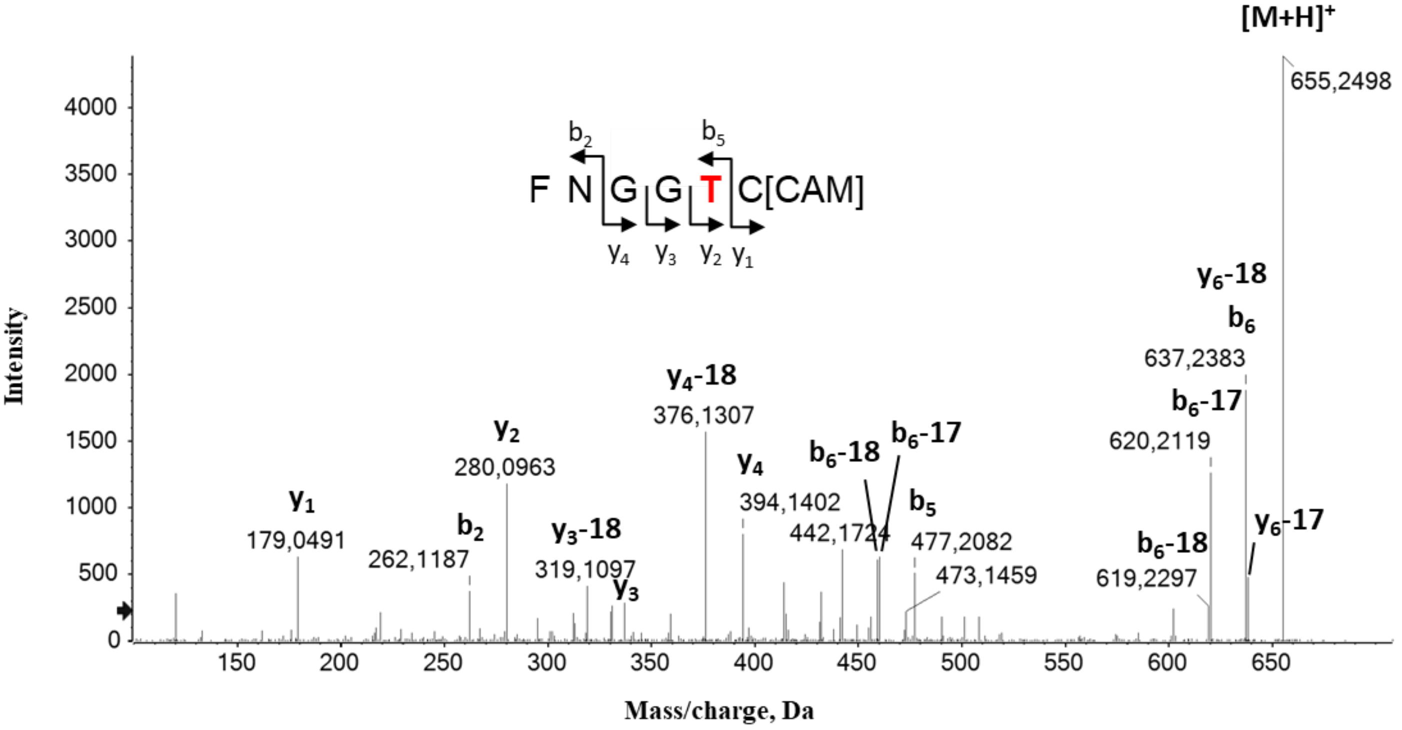

B.

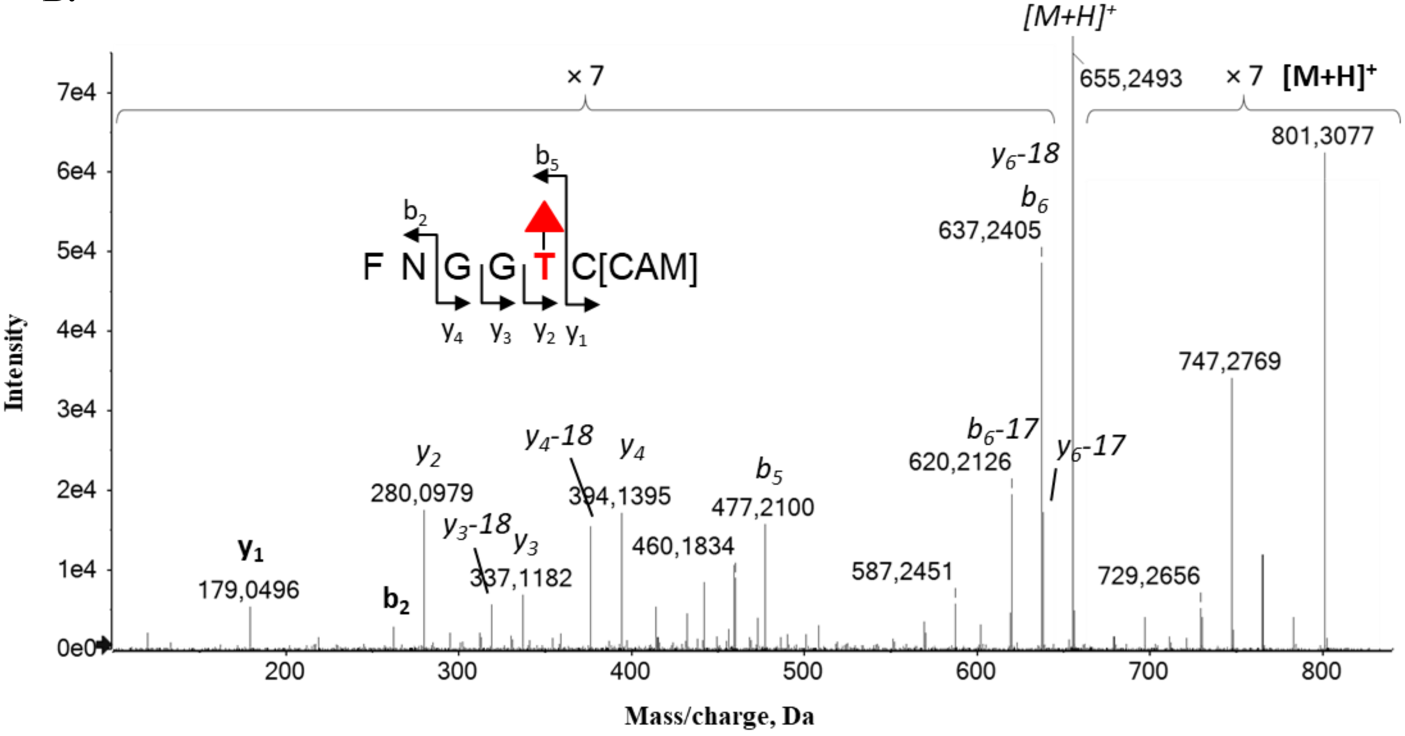

Figure S3. Representative MS/MS spectra of the peptide of interest from WT EGF-LD III co-digested with trypsin and thermolysin, after incubation with POFUT1 and GDP-fucose. Spectra, acquired with a micro-LC TripleTOF system in IDA mode, are shown for EGF-LD III peptide, non-modified WT (**A**) and modified with *O*-fucose (**B**). Detected b/y fragments are annotated on each spectrum confirming the identified peptide sequence. The presence of non-modified y fragments, indicated in italic (B), reflects the loss of *O*-fucose moeity which had been transferred. The threonine T^{255} of the *O*-fucosylation consensus site is indicated in red bold.

SUPPLEMENTARY DATA

Targeted sequence	charge state	Non-modified peptide MS1	Non-modified peptide MS2		Modified peptide MS1	Modified peptide MS2		DP (V)	CE (V)
**WT EGF-LD III**	+3	681.2782	+3y10	1100.4829	729.9642	+3y10	1246.5408	80.8	34.7
ANC[CAM]STTC[CAM]FNGG**T**C[CAM]FYPGK		detected at	+3y9	986.4400	detected at	+3y9	1132.4979		
		RT 9.73 min	+3y6	771.34942	RT 9.57 min	+3y6	771.3494		
			+3y4	464.2503		+3y4	464.2503		
			**+3y3**	**301.1870**		**+3y3**	**301.1870**		
			+3b4	433.1499		+3b4	433.1499		
**T255A EGF-LD III**	+3	671.2747	+3y10	1070.4723	719.9607	+3y10	1216.5303	80.1	34.1
ANC[CAM]STTC[CAM]FNGG**A**C[CAM]FYPGK		detected at	+3y9	956.42946	not detected	+3y9	1102.4873		
		RT 9.76 min	+3y8	899.4080		+3y8	1045.4659		
			+3y6	771.3494		+3y6	771.3494		
			+3y4	464.2503		+3y4	464.2503		
			**+3y3**	**301.1870**		**+3y3**	**301.1870**		

Table S1. MRM^{HR} parameters for the detection of WIF1 EGF-LD III WT and mutated peptides. The *O*-fucosylation site is indicated in bold and underlined. Each peptide is detected by a single charge state corresponding to a MS1 value (with optimized Declustering Potential (DP) and collision energy (CE)) recorded during the 30 min analysis. Peak detection with MultiQuant was achieved with the six most abundant fragments (MS2) with a 10,000 resolution. Quantification fragment is indicated in bold. The 5 other MS2 were used as confirmation fragments.

Targeted sequence	charge state	Non-modified peptide MS1	Non-modified peptide MS2		Modified peptide MS1	Modified peptide MS2		DP (V)	CE (V)
**WT EGF-LD V**	+4	770.0887	+4y8	942.4097	806.6032	+4y8	1088.4677	87.3	39.5
MDIGINSDPKPVC[CAM]EPGC[CAM]GAHG**T**		detected at	**+4y3**	**358.2084**	not detected	**+4y3**	**358.2084**		
C[CAM]HEPNK		RT 12.17 min	+4y20+2	1116.4907		+4y20+2	1189.5197		
			+4y18+2	1003.9169		+4y18+2	1076.9458		
			+4y14+2	761.3197		+4y14+2	834.3486		
			+4b2	247.0747		+4b2	247.0747		
**T319A EGF-LD V**	+4	762.5861	+4y8	912.3992	799.1006	+4y8	1058.4571	86.7	39.1
MDIGINSDPKPVC[CAM]EPGC[CAM]GAHG**A**		detected at	**+4y3**	**358.2084**	not detected	**+4y3**	**358.2084**		
C[CAM]HEPNK		RT 12.27 min	+4y20+2	1101.4855		+4y20+2	1174.5144		
			+4y18+2	988.9116		+4y18+2	1061.9406		
			+4y14+2	746.3144		+4y14+2	819.3433		
			+4b2	247.0747		+4b2	247.0747		

Table S2. **MRM^{HR} parameters for the detection of WIF1 EGF-LD V WT and mutated peptides**. The *O*-fucosylation site is indicated in bold and underlined. Each peptide is detected by a single charge state corresponding to a MS1 value (with optimized Declustering Potential (DP) and collision energy (CE)) recorded during the 30 min analysis. Peak detection with MultiQuant was achieved with the six most abundant fragments (MS2) with a 10,000 resolution. Quantification fragment is indicated in bold. The 5 other MS2 were used as confirmation fragments.

Targeted sequence	charge state	Non-modified peptide MS1	Non-modified peptide MS2		Modified peptide MS1	Modified peptide MS2		DP (V)	CE (V)
**WT EGF-LD III**	+1	655.2504	$[M+H]^+$	655.2504	801.3083	$[M+H]^+$ -Fuc	655.2504	78.9	33.8
FNGG**T**C[CAM]		detected at	+1y6-18	637.2399	detected at	+1y6-18 -Fuc	637.2399		
		RT 8.83 min	+1y4-18	376.1285	RT 8.83 min	+1y4-18 -Fuc	376.1285		
			+1y2	280.0961		+1y2 -Fuc	280.0961		
			+1y4	394.1391		+1y4 -Fuc	394.1391		
			+1b6-17	620.2133		+1b6-17 -Fuc	620.2133		

Table S3. MRM^{HR} parameters for the detection of WIF1 EGF-LD III WT peptide, obtained after co-digestion with trypsin and thermolysin. The *O*-fucosylation site is indicated in bold and underlined. Each peptide is detected by a single charge state corresponding to a MS1 value (with optimized Declustering Potential (DP) and collision energy (CE)) recorded during the 30 min analysis. Peak detection with MultiQuant was achieved with the six most abundant fragments (MS2) with a 10,000 resolution for non-modified. Fragments corresponding to the loss of the sugar moiety were added for modified peptide.

SUPPLEMENTARY DATA

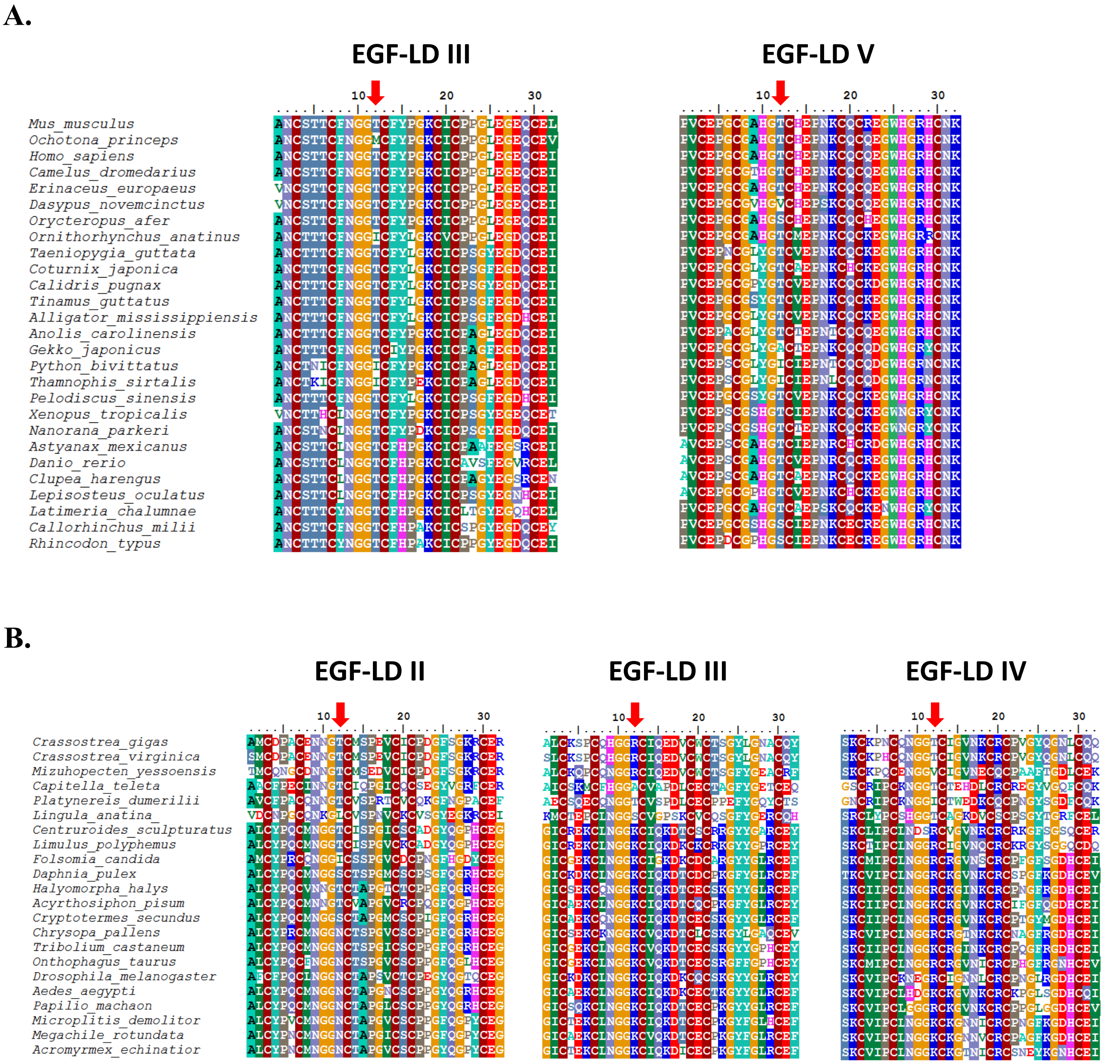

Figure S4. Alignment of the Wif1 EGF-LDs with an *O*-fucosylation site among Gnathostomes (A) and Protostomes (B). Red arrows point the threonine or serine potentially modified with *O*-fucose, included in the consensus sequence $C^2XXXX(\underline{S}/\underline{T})C^3$.

SUPPLEMENTARY DATA

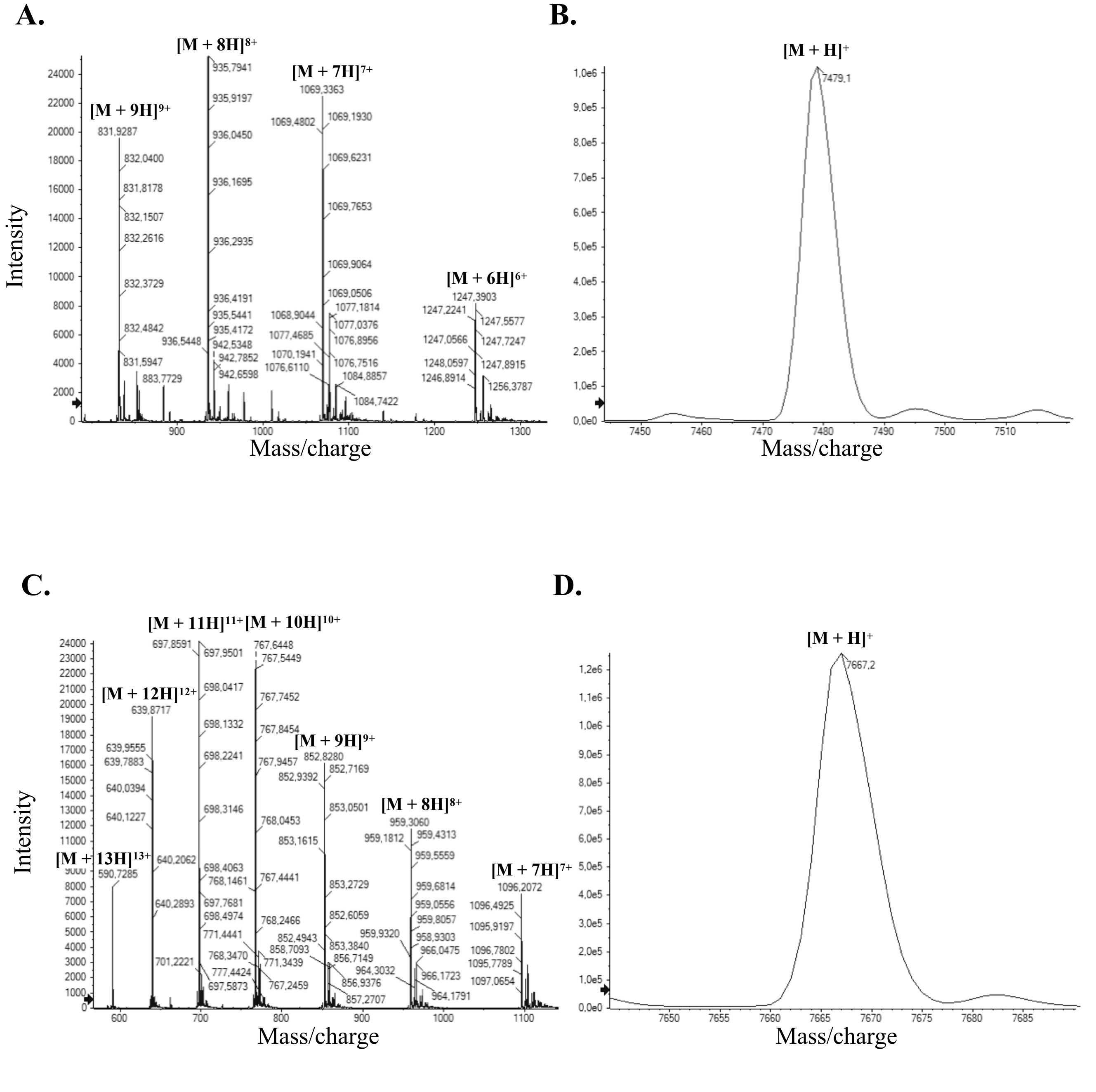

Figure S5. **ESI-TOFMS spectra of isolated EGF-LD III (A, B) and EGF-LD V (C, D) of mouse WIF1.** Detected signals correspond to multi-charged forms ($[M + 9H]^{9+}$ to $[M + 6H]^{6+}$) of EGF-LD III (A) and to multi-charged forms ($[M + 13H]^{13+}$ to $[M + 7H]^{7+}$) of EGF-LD V (C). Deconvoluted ESI mass spectra of purified WIF1 EGF-LD III (B) and EGF-LD V (D) suggest the presence of three disufilde bonds comparing to the theorical molecular weight.

SUPPLEMENTARY DATA

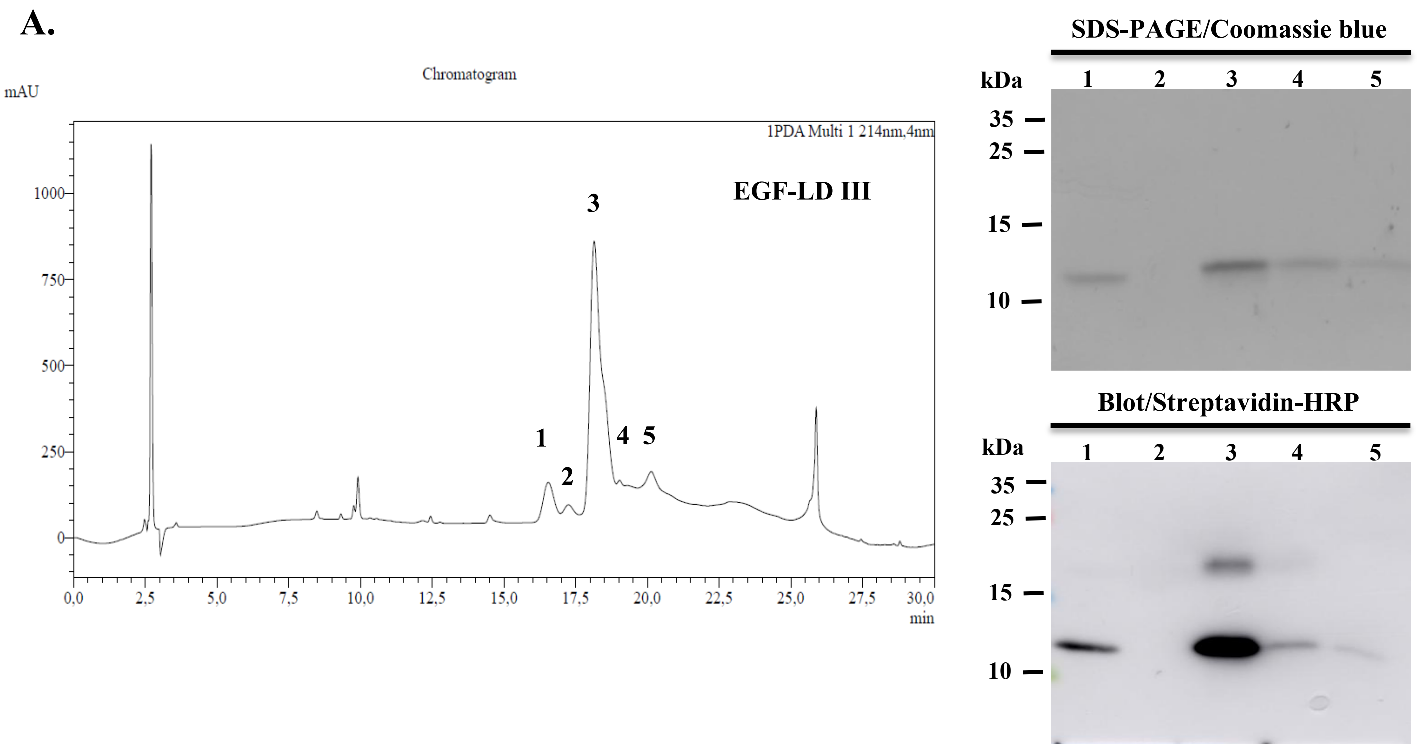

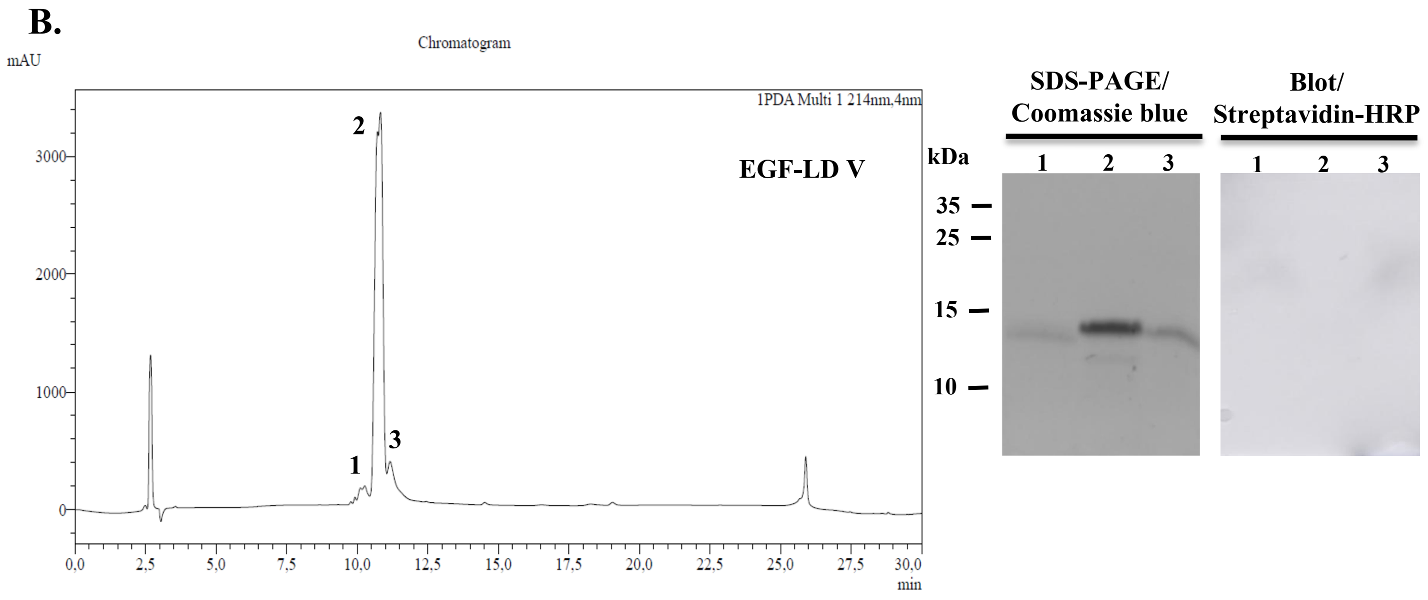

Figure S6. Reverse-phase HPLC of Ni-NTA purified WT WIF1 EGF-LD III (A) and EGF-LD V (B). A major peak was detected for both EGF-LDs and additional minor peaks. Numbered peaks were collected and proteins analyzed by SDS-PAGE and by Blot/streptavidin-HRP after *in vitro O*-fucosylation and click chemistry. For EGF-LD III, all folding isomers detected on blue-stained gel were modified with *O*-fucose. For EGF-LD V, no isomer was modified as previously shown.

SUPPLEMENTARY DATA

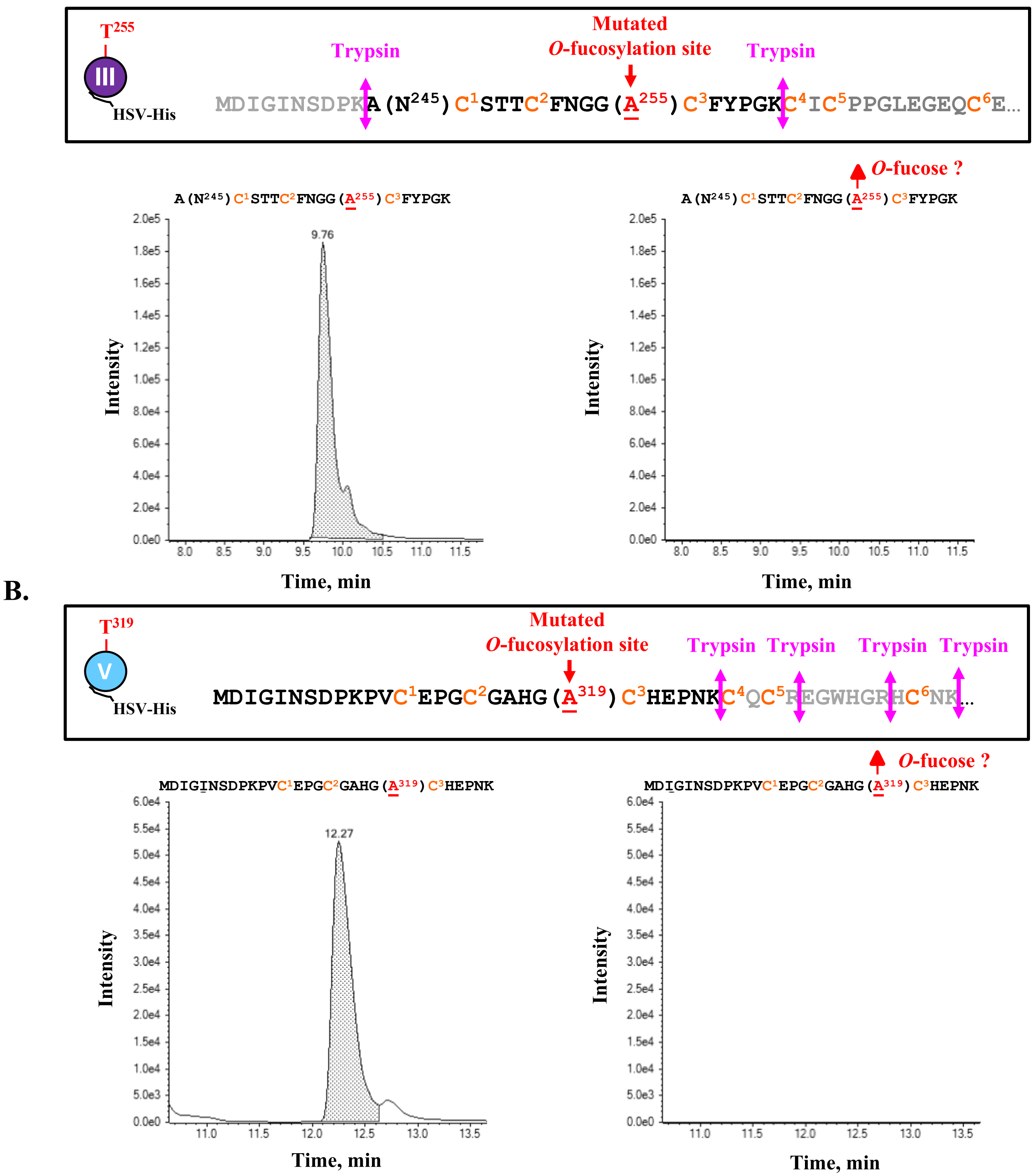

Figure S7. MRM-MS analysis of trypsin-digested isolated T/A mutated EGF-LDs III and V of mouse WIF1, after an *in vitro O*-fucosylation assay. **(A)** T255A EGF-LD III and **(B)** T319A EGF-LD V, produced and purified from *E. coli* BL21 strain, were first independently incubated with recPOFUT1 and GDP-fucose to allow *in vitro O*-fucosylation. After reduction, alkylation and trypsin digestion of these glycosyltransferase reactions, resulting peptides were analyzed by micro-LC MRM-MS. Non-modified peptides were detected for mutated EGF-LDs III and V (left panels). No peptide modified with *O*-fucose was detected when the *O*-fucosylation sites were mutated in alanine (T255A, T319A)(right panels).

The amino acid sequence of each WIF1 EGF-LD is indicated with its mutated *O*-fucosylation consensus site in red and between brackets. The six conserved cysteines involved in disulfide bonds are numbered and trypsin cleavage sites are shown with pink double arrows. A potential *N*-glycosylation site is shown (N^{245}).

SUPPLEMENTARY DATA

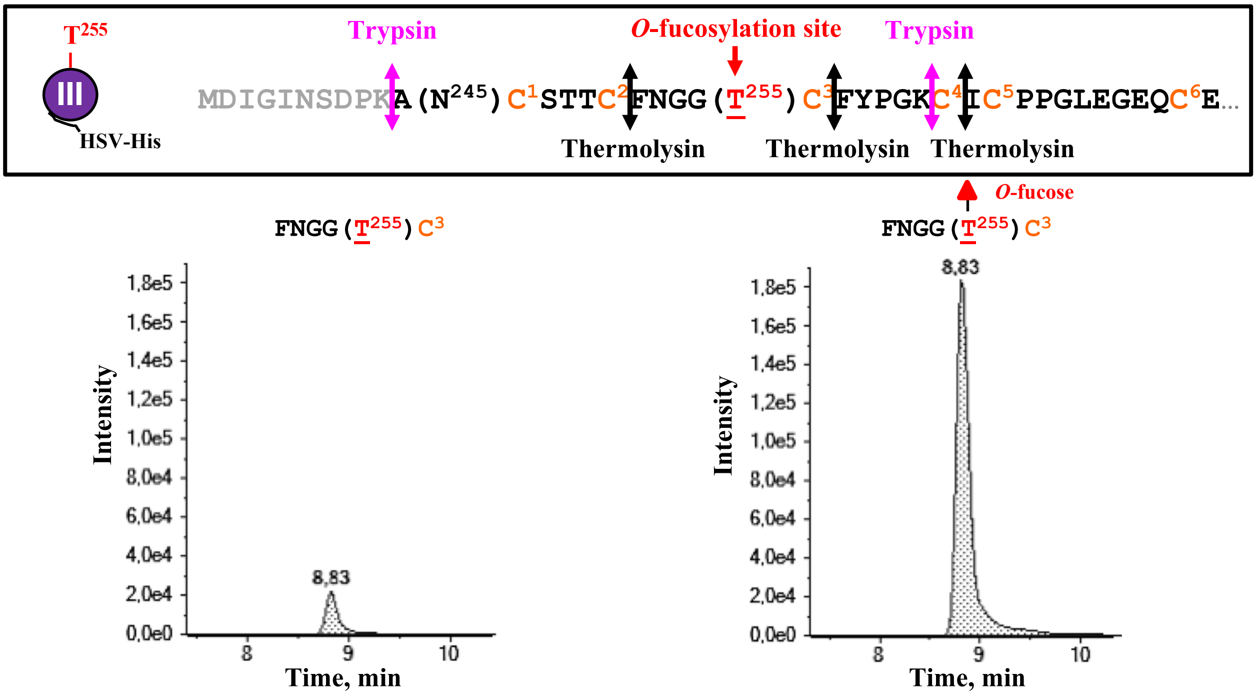

Figure S8. MRM-MS of EGF-LD III co-digested with trypsin and thermolysin, after an *in vitro O*-fucosylation assay. The amino acid sequence of WIF1 EGF-LD III is indicated with its *O*-fucosylation consensus site in red and between brackets. Conserved cysteines are numbered and trypsin and thermolysin cleavage sites within EGF-LD are shown with pink and black double arrows, respectively. WIF1 EGF-LD III was incubated with recPOFUT1 and GDP-fucose to allow *in vitro O*-fucosylation. After reduction, alkylation and trypsin/thermolysin co-digestion, resulting peptides were analyzed by micro-LC MRM-MS. Both peptides, with no modification (**left panel**) and with *O*-fucose (**right panel**), were detected for EGF-LD III.

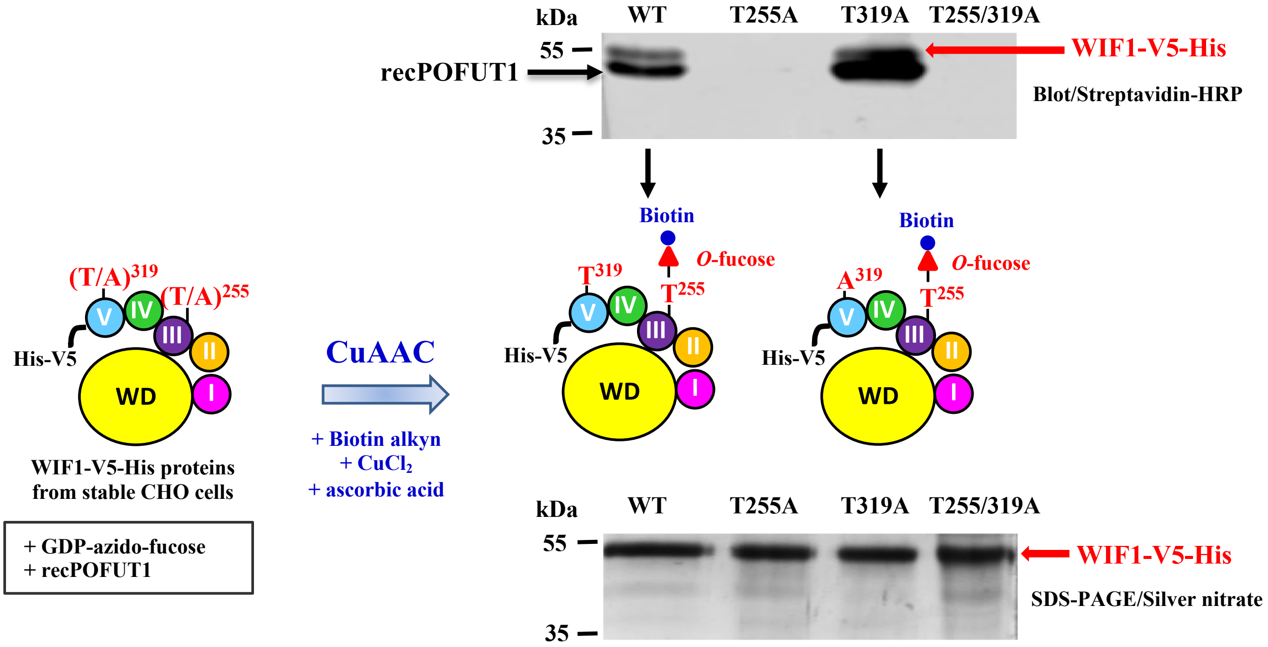

Figure S9. Chemoenzymatic approach to reveal ability of recombinant mouse WIF1-V5-His to receive *O*-fucose. WT and mutated (T255A, T319A and T255/319A) variants for mouse WIF1-V5-His were purified from stable Flp-In^{TM} CHO cells and subjected to recPOFUT1 and GDP-azido-fucose. Click chemistry (CuAAC) was performed using alkynyl biotin to covalently attach biotin to fucose if transferred to WIF1-V5-His protein by recPOFUT1. After separation by SDS-PAGE and protein transfer, protein biotinylation was detected using streptavidin-HRP. Positive signals corresponded to successful *in vitro* POFUT1-mediated azido-fucose transfer to recombinant WIF1-V5-His proteins (upper panel), for which quantity and purity were checked by silver nitrate-stained polyacrylamide gels (lower panel). RecPOFUT1, which remains bound to untransferred azido-fucose, appeared labelled at around 45 kDa after incubation with WT or T319A WIF1.

SUPPLEMENTARY DATA

A.

EGF-LD III peptide containing the *O*-fucosylation site
FNGG**T**C[CAM]

	Non-modified form		Elongated forms									
**Searched generic *O*-sugar and elongation**			-dHex		-dHex-HexNac		-dHex-HexNAc-Hex		-dHex-HexNAc-Hex-NeuAc		-dHex-HexNAc-Hex-NeuGc	
**Proposed *O*-sugar and elongation**			Fuc		Fuc–GlcNAc		Fuc–GlcNAc–Gal		Fuc–GlcNAc–Gal–Neu5Ac		Fuc–GlcNAc–Gal–Neu5Gc	
**MS1 Precursor detection**	**theorical mass**	**observed mass**	**theorical mass**	**observed mass**	**theorical mass**	**observed mass**	**theorical mass**	**observed mass**	**theorical mass**	**observed mass**	**theorical mass**	**observed mass**
	655.2504	655.2505	801.3083	801.3100	1004.3877	not detected	1166.4405	not detected	723.2716	not detected	737.2691	not detected
	error: 0.2 ppm		error: 2.1 ppm									

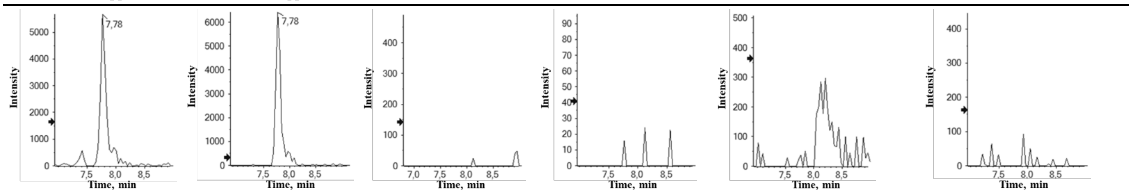

MS2
Peptide fragment detection

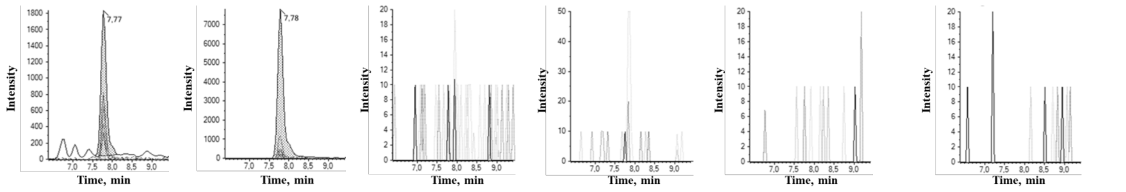

B.

EGF-LD V peptide containing the *O*-fucosylation site
VC[CAM]EPGC[CAM]GAHG**T**C[CAM]HEPNK

	Non-modified form		Elongated form	
**Searched generic *O*-sugar and elongation**			-dHex	
**Proposed *O*-sugar and elongation**			Fuc	
**MS1 Precursor detection**	**theorical mass**	**observed mass**	**theorical mass**	**observed mass**
	478.1989	478.1987	514.7134	not detected
	error: -0.4 ppm			

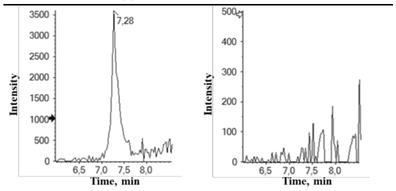

MS2
Peptide fragment detection

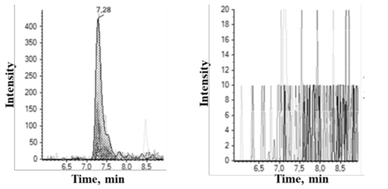

Figure S10. Mass spectrometry data of elongated forms for *O*-fucose carried by WIF1-V5-His, after co-digestion with trypsin and thermolysin. For EGF-LD III peptide (the *O*-fucosylation site is indicated in bold red), *m/z* were calculated for each possible elongated form of the *O*-deoxyhexose carried by T^{255} (namely an *O*-fucose) and searched in MS1 spectra from MRM-MS data. When found, observed *m/z* was reported for the precursor ion and an error was calculated. Ion spectra for each elongated form precursor were extracted even if no peaks were detected at the suspected retention time. A specific MRM-MS method was created with all the elongated forms for EGF-LD III peptide and also with non-modified EGF-LD V peptide or modified with an *O*-deoxyhexose. MRM^{HR} parameters for the detection of elongated form fragments were the same as the monosaccharide form for EGF-LD III peptide (Supplementary Data Table 3).

(A) For **EGF-LD III**, the mainly detected elongated form corresponds to an *O*-deoxyhexose added, which must be an *O*-fucose. The non-modified form was detected in both MS1 (upper panels) and MS2 (lower panels) data but all combinations of peptides with elongated *O*-fucose were not detected in MS1 or in MS2.

(B) For **EGF-LD V,** only the non-modified form was detected.

<u>Symbols used</u>: Fucose (Fuc) (red filled triangle), Galactose (Gal) (yellow filled circle), *N*-acetylglucosamine (GlcNAc) (blue filled square), *N*-acetylneuraminic acid (Neu5Ac) (purple filled diamond), *N*-glycolylneuraminic acid (Neu5Gc) (light blue filled diamond).

SUPPLEMENTARY DATA

Daphnia pulex

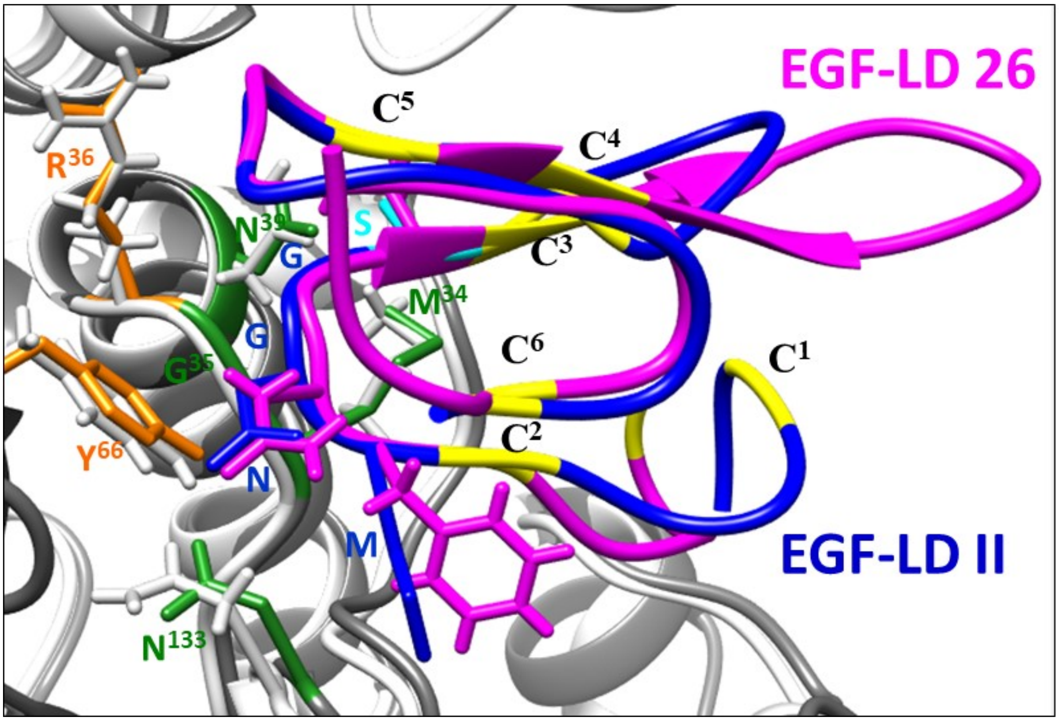

>*Daphnia pulex*_EFX65761.1_EGF-LD II

ALCYPQCMNGGSCTSPGMCSCPSGFQGRHCEG

Figure S11. **Automatic models for Wif1 EGF-LD II and Pofut1 of *Daphnia pulex (Dp)*.** Using Matchmaker, structural automated models for *Dp* Pofut1 (EFX81920.1, 381 aa) (dim gray) and for *Dp* Wif1 EGF-LD II (EFX65761.1_EGF-LD II) (blue) were superimposed to mouse POFUT1 (light gray) / N1-EGF-LD 26 (magenta) complexes (PDB5KY4). Key residues (green) of *Dp* Pofut1 matched with those of mouse Pofut1, known to be involved in interaction with the C^2-C^3 subdomain of N1-EGF-LD 26. Side chains of residues are shown for the C^2-C^3 subdomains and residues of interest. No steric clash was found.
